# The transcriptional integration of environmental cues with root cell type development

**DOI:** 10.1093/plphys/kiae425

**Published:** 2024-09-17

**Authors:** Mona Gouran, Siobhan M Brady

**Affiliations:** Department of Plant Biology and Genome Center, UC Davis, Davis, CA 95616, USA; Department of Plant Biology and Genome Center, UC Davis, Davis, CA 95616, USA

## Abstract

Plant roots navigate the soil ecosystem with each cell type uniquely responding to environmental stimuli. Below ground, the plant's response to its surroundings is orchestrated at the cellular level, including morphological and molecular adaptations that shape root system architecture as well as tissue and organ functionality. Our understanding of the transcriptional responses at cell type resolution has been profoundly enhanced by studies of the model plant *Arabidopsis thaliana*. However, both a comprehensive view of the transcriptional basis of these cellular responses to single and combinatorial environmental cues in diverse plant species remains elusive. In this review, we highlight the ability of root cell types to undergo specific anatomical or morphological changes in response to abiotic and biotic stresses or cues and how they collectively contribute to the plant's overall physiology. We further explore interconnections between stress and the temporal nature of developmental pathways and discuss examples of how this transcriptional reprogramming influences cell type identity and function. Finally, we highlight the power of single-cell and spatial transcriptomic approaches to refine our understanding of how environmental factors fine tune root spatiotemporal development. These complex root system responses underscore the importance of spatiotemporal transcriptional mapping, with significant implications for enhanced agricultural resilience.

## Introduction

Plant roots, often buried deep in the soil, are composed of multiple cell types that collectively form an organ that provides nutrients and support for plant growth and development. These hidden structures perform many essential functions, including providing mechanical stability and facilitating the absorption of water and nutrients from the soil and transporting them into above-ground tissues. Roots also serve as the gateway to interactions with the surrounding complex soil environment known as the rhizosphere. Here, roots are constantly challenged by a variety of biotic and abiotic stimuli. Biotic interactions include beneficial symbiotic associations with mycorrhizal fungi and nitrogen-fixing bacteria and potentially detrimental encounters with pathogens, parasites, and herbivores. Abiotic stressors, in contrast, arise from nonliving factors such as drought, salinity, extreme temperatures, and soil contaminants. Root architecture and anatomy are dynamically modulated by these environmental cues. These changes often represent adaptive strategies aimed at enhancing the plant's chances of survival. The molecular basis for this developmental plasticity includes cellular reprogramming of cell populations, which result in the production of an optimal root system to face a given environmental perturbation.

The most characterized root at the cellular and transcriptional level is that of *Arabidopsis thaliana* due to its simplicity in developmental patterning. The root stem cell niche gives rise to 5 different tissues that form the majority of the root and that are largely patterned with radial symmetry. The outermost tissue is the epidermis, composed of hair cells (trichoblasts) and nonhair cells (atrichoblasts), followed by 1 (in Arabidopsis) and up to several layers of cortex cells; the endodermis; pericycle (including xylem pole pericycle and phloem pole pericycle cells); as well as vascular tissue, which is comprised of xylem, phloem, and procambium. Vascular cells have diverse patterning dependent on the species. The stele is comprised of pericycle and vascular tissue. Along the root's longitudinal axis, cell types undergo development in 3 developmental zones. The meristematic zone consists of rapidly proliferating cells. Cells then transition into the elongation zone, where they expand in size. Ultimately, cells progress into the differentiation zone, where they acquire their final developmental characteristics required to carry out their respective functions. To adapt to challenges in their underground environment, each of the root cell layers functions as an environmental sensor, and as such, the development of each cell type is interdependent with its surroundings. Each cell type relies on a complex gene regulatory network that is finely calibrated by environmental signals. These sophisticated networks govern cell type–specific adaptations to both abiotic and biotic stresses. Bulk transcriptomic studies have provided insight in understanding root system plasticity at the tissue and organ level. While several studies have explored cell type resolution transcriptional responses to specific external factors, there still remains a significant gap in systematic elucidation of mechanisms underlying cell type–specific transcriptional reprogramming in response to single and combinatorial stresses. Bridging this gap necessitated the development of methodologies for higher-spatiotemporal resolution profiling that is amenable to different plant species. Recent technological advancements that enable transcriptome surveys at single-cell resolution have begun to close this gap. Of particular interest are cases where they are used to interrogate how environmental stressors impact cell identities and states. Elaboration of such single-cell omics datasets to include stress responsiveness at cellular resolution in crops are particularly important to inform cell and tissue-specific targets to enhance stress resilience with minimal undesirable pleiotropic effects.

This review aims to provide an overview of how different plant root cell types respond to various abiotic and biotic stimuli. By examining cell type–specific anatomical, morphological, and transcriptional changes, we highlight the dynamic nature of these responses. We also explore advancements in single-cell and spatial transcriptomic approaches that offer new insights into these processes, emphasizing their implications for understanding plant–environment interactions.

### Root cell type–specific adaptive responses to environment

**Figure 1.**
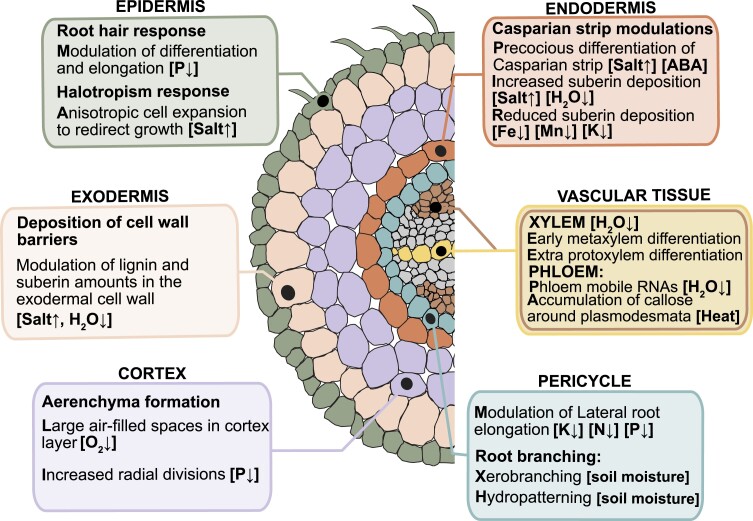
Root cell type–specific anatomical and morphological responses to stresses. Schematic representation of a plant root cross-section highlighting modes of adaptations of different root cell layers to select environmental factors.

Root cellular morphology and cell wall composition vary between different cell types in an individual plant and between species. This inherent variability primes each cell type to display a unique and tailored response to the environment throughout development. Plants adapt to unique and diverse environments; thus, evolution has likely shaped a multitude of cellular strategies. As the outermost root cell layer, epidermal cells play a pivotal role as the primary interface with the soil environment (Fig. 1). Epidermal root hairs are specialized single-celled cylindrical projections of the epidermis (Cormack 1949; Salazar-Henao et al. 2016) that are strongly responsive to environmental factors. To optimize nutrient ion and water uptake, root hair specification and elongation are modulated in response to available soil resources. Notably, these include mineral nutrients with low mobility in most soil systems, including inorganic phosphate (Pi) (Bates and Lynch 1996), nitrogen (N), calcium (Ca), sulfur (S), sodium (Na) (Libault et al. 2010; Salazar-Henao et al. 2016), and manganese (Mn) (Yang et al. 2008), as well as fluctuations in temperature (Fan et al. 2022). The mode of root hair cellular differentiation response is matched to the type of stress the root experiences, depending on the species. Root hair specification and elongation, for example, is stimulated in low-phosphate conditions in Arabidopsis, tomato, maize, and citrus (Bates and Lynch 1996; Zhu et al. 2005; Cao et al. 2013; Demirer et al. 2023) while under salt stress, both these developmental processes are suppressed in Arabidopsis and rice (Wang et al. 2008; Robin et al. 2016). Epidermal cells are also responsible for detecting and initiating subsequent signal transduction processes. An example of this is root halotropism or “salt-avoidance” (Sun et al. 2008). Under salt stress (NaCl), plant roots can override their gravitropic responses through anisotropic epidermal cell expansion (Yu et al. 2022). This is facilitated by auxin redistribution via salt-induced endocytosis of PIN-FORMED (PIN2) auxin carrier proteins in the epidermal cell membrane facing the higher salt concentrations (Galvan-Ampudia et al. 2013), accompanied by microtubule reorientation that guides microfibril deposition pattern (Yu et al. 2022). These coordinated epidermal-specific changes redirect growth of roots away from the high-salt environment.

The exodermal layer is located underneath the epidermis and is considered the outermost cortex layer (Kajala et al. 2021) (Fig. 1). This cell type has gained less attention in recent decades as it is absent in the model plant Arabidopsis but is present in most angiosperms (Perumalla et al. 2008). Similar to the endodermis, the exodermis is reported to contain both lignified and suberized cell walls (Perumalla et al. 2008; Kajala et al. 2021; Manzano et al. 2022; Cantó-Pastor et al. 2024). These chemical structures in the exodermal cell wall are largely presumed to act as apoplastic barriers, regulating radial water and solute transport in the root (Hose et al. 2001; Enstone et al. 2002). Exodermal barriers are highly responsive to extreme environments through modifications in the amount of these polymers deposited under stress (Soukup et al. 2007; Shao et al. 2021; Cantó-Pastor et al. 2024). Interestingly, certain plant species lacking an exodermis under optimal growth conditions can deposit suberin lamellae in the outer cortex cell layer in response to stress as seen in cotton and barley under high salt and osmotic stress, respectively (Reinhardt and Rost 1995; Kreszies et al. 2020).

Extensive variation in the number of cortex cell files is present within and between plant species. While Arabidopsis is composed of a single cortex layer, many crop species, including maize, rice, and tomato, have multiple cortex cell files (Rebouillat et al. 2009; Burton et al. 2013; Ron et al. 2013). Anatomical differences in the number and size of the cortical cells are associated with a variety of beneficial physiological adaptations in plants. For example, larger cortical cell size in maize genotypes is associated with decreased root respiration, increased rooting depth, and enhanced water uptake under water-limiting conditions (Chimungu et al. 2014a, 2014b). Beyond anatomical differences, cortical differentiation programs display plasticity in response to growth conditions. In Arabidopsis, low phosphate levels trigger increased radial divisions in the cortex layer, leading to a greater number of cortical cell files and thus more cortical cell junctions. This presumably causes more epidermal cells to receive the positional cue for trichoblast fate, resulting in higher root hair density (Cederholm and Benfey 2015). Cortex cells also undergo aerenchyma or air space formation. Under anoxic conditions, aerenchyma formation is induced as cortex cells undergo programmed cell death (lysigenous aerenchyma), creating large air-filled spaces in many crop species (Drew et al. 2000; Nishiuchi et al. 2012). This increased air space facilitates gas exchange and oxygen diffusion to the submerged parts of the root. Maize genotypes with increased aerenchyma are also associated with drought tolerance (Zhu et al. 2010). In addition to its formation in anoxic conditions, in wheat, root cortical aerenchyma is also induced in response to soil compaction (Fig. 1) (Colombi and Walter 2017).

Surrounding the stelar tissue is the endodermis (Fig. 1). Endodermal differentiation involves cell wall modifications in the form of the Casparian strip and suberin lamellae. The Casparian strip is a lignin-rich structure deposited in a discrete domain along the central axis of endodermal cells, which acts as an apoplastic barrier from the cortex into the central vascular tissue and vice versa (Alassimone et al. 2010; Naseer et al. 2012). Following Casparian strip synthesis and deposition, hydrophobic suberin lamellae are deposited on the entire cell surface, creating a diffusion barrier for the transcellular pathway (Robbins et al. 2014; Andersen et al. 2015; Shukla et al. 2021). Similar to the exodermis, external factors influence the development of the endodermal Casparian strip and suberin lamellae. These include salt and drought, which, through the plant hormones abscisic acid (ABA) and ethylene, regulate the biosynthesis and degradation of endodermal suberin lamellae in response to nutrient stress (Barberon et al. 2016). For instance, in response to salt stress, the Casparian strip matures earlier in Arabidopsis, cotton, and maize endodermal cells (Reinhardt and Rost 1995; Karahara et al. 2004; Barberon et al. 2016). Collectively, these cellular differentiation features constitute physiologically relevant responses, mediated by endodermal cells, that contribute to overall root function in different environments.

The pericycle is the outermost radial cell layer surrounding the vascular cylinder (Fig. 1). This cell type is unique in that it retains pluripotency and hence can continuously form new tissues. In Arabidopsis, a few prepatterned pericycle cells known as “founder cells” adjacent to the xylem poles are sites of lateral root initiation and emergence (although not applicable to all vascular plants), commonly known as root branching (De Smet et al. 2006; Moreno-Risueno et al. 2010; Santos Teixeira and Ten Tusscher 2019). Lateral root formation is a key developmental mechanism to increase the root system's surface area, thereby enhancing its adaptability to the soil environment. External factors—such as soil moisture and nutrient availability, including nitrogen, potassium, and phosphate—impact the process of root branching (Zhang and Forde 2000; Armengaud et al. 2004; Miura et al. 2011). Arabidopsis seedlings cease lateral root elongation in potassium-deficient media, whereas low nitrogen and phosphate promote lateral root formation and elongation to scavenge available soil resources (Zhang and Forde 2000; Armengaud et al. 2004; Pérez-Torres et al. 2008; Miura et al. 2011; Pélissier et al. 2021). Local repression of lateral root initiation is observed in cereal crops (maize and barley), as well as Arabidopsis, when the root is exposed to small air macropores in soil environment, inducing a transient and local water deficit. This adaptive response at a macroscale is known as xerobranching and is mediated by ABA signaling and auxin (Orman-Ligeza et al. 2018).

Deep within the root is the vascular tissue, a transport system composed of 2 functionally distinct cell types and tissues: xylem and phloem, and their stem cells collectively termed the procambium (Fig. 1). Xylem cells are composed of 2 types: proto- and metaxylem. Protoxylem are developed earlier in root development and are characterized by spiral, helical, or annular secondary cell wall thickenings. Metaxylem develop later with characteristic pitted and heavily lignified secondary cell walls, which are dead at maturity and resemble hollow tubes (Kubo et al. 2005; Růžička et al. 2015). Water and minerals are transported through xylem cells from the root to the shoot. Conversely, phloem distributes photosynthate from “source” to “sink” tissues (Lucas et al. 2013). Xylem differentiation is plastic and responds to environmental cues such as water limitation and salinity. In Arabidopsis, under water deficit stress, metaxylem differentiates closer to the root tip, while extra protoxylem cell files differentiate in the root tip. These developmental responses are mediated by ABA-induced regulation of miR165, which ultimately regulates class III homeodomain leucine zipper transcription factors transcript abundance, resulting in protoxylem specification instead of metaxylem (Ramachandran et al. 2018, 2021; Bloch et al. 2019). In several dicot species salinity inhibits local protoxylem differentiation via a DELLA-mediated repression of gibberellic acid signaling. This reduction promotes expression of the xylem master regulator *VASCULAR NAC_DOMAIN 6* (*VND6*), leading to the discontinuous formation of protoxylem cell files. Notably, this developmental response is correlated with salt tolerance (Augstein and Carlsbecker 2022). The plasticity of vascular system differentiation in response to external stimuli is also evident during secondary growth in woody species. For instance, *Populus* produces xylem vessel elements with narrow lumens under drought to mitigate cavitation and hydraulic failure (Rodriguez-Zaccaro and Groover 2019; Rodriguez-Zaccaro et al. 2021).

Similar to the role of xylem cells in transporting water and minerals, sieve elements within phloem tissue are crucial for distributing photosynthates and nutrients to the developing tissues, as well as delivery of intracellular and long-distance signals, which is required for systemic adaptation to stress conditions (Ham and Lucas 2014). Shoot-derived mobile RNAs mediate plant responses to abiotic stresses through phloem transport (Liu et al. 2023). Phloem cells can also change their structure in response to stress. During heat stress, phloem unloading is modulated by accumulation of callose around plasmodesmata at the junctions between sieve elements and phloem pole pericycle. This accumulation restricts the flow through plasmodesmata, reducing phloem unloading activity and subsequent inhibition of root growth (Liu et al. 2022b). Thus, from the outermost epidermal cells to the inner vascular tissue, each root cell type undergoes dynamic, diverse, and specialized responses to environmental cues to optimize their function to mitigate environmental challenges (Fig. 1).

### Root cell type–specific transcriptional dynamics in response to the environment

**Figure 2.**
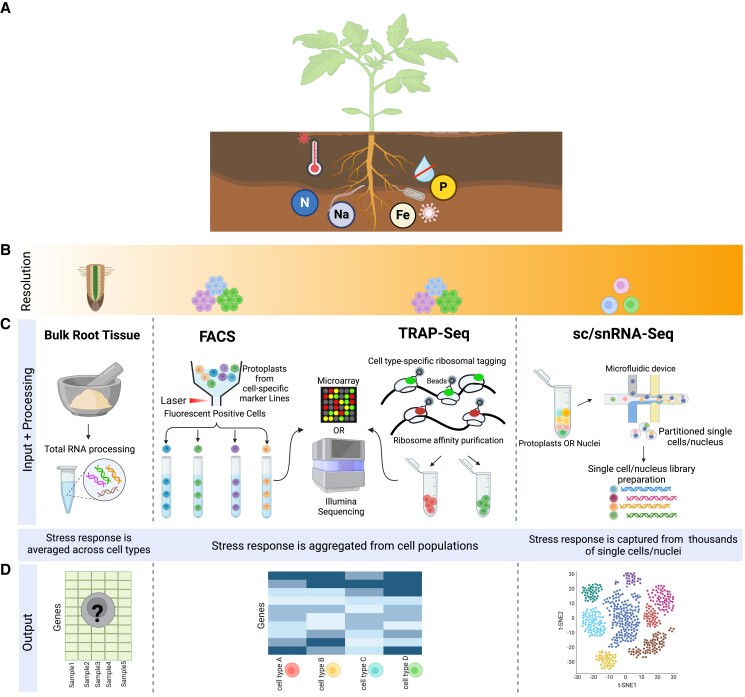
Overview of transcriptomic approaches used for analysis of root stress response. **A)** Root system facing multiple biotic and abiotic stressors in the soil environment. **B)** Increasing resolution of transcriptomic methods. The progression moves from the entire root tissue, through intermediate resolutions examining specific cell populations, to the highest resolution of sc/snRNA-Seq, capturing individual cells. **C)** Workflow illustrating the processing for different techniques. Bulk root tissue analysis processes total RNA yielding an average stress response across all cell types. FACS: Allows for the isolation of specific cell types based on fluorescence markers. Advantage: High specificity in sorting. Disadvantage: Requires fluorescent markers and cell dissociation, which can alter gene expression. TRAP-Seq pools multiple cells of a single type. Advantage: Focuses on actively translated genes. Disadvantage: Requires marker lines expressing tagged ribosomes. sc/snRNA-Seq involves partitioning individual cells/nuclei using a microfluidic device to capture a detailed stress response profile from each cell. scRNA-Seq advantage: Provides a comprehensive transcriptome profile of individual cells, capturing both nuclear and cytoplasmic. Disadvantage: Requires cell dissociation, which can induce stress responses. It can also be challenging to isolate specific plant cell types due to the rigid cell walls or their size. snRNA-Seq advantage: Bypasses the need for cell dissociation, preserving the transcriptional state without the stress of cell wall digestion. This method is particularly useful for fixed or frozen samples and for plant cells with rigid secondary cell walls. **D)** Comparisons of the resolution of gene expression data obtained from the various transcriptome techniques. Figure created with Biorender.com.

Every cell type in a plant root has the same genetic makeup, yet they develop unique phenotypes in response to various environmental stimuli. Traditionally, these cell type–specific phenotypes have been studied by examining cell structure and form. It is important to note, however, that lack of a morphological or observable phenotype does not necessarily indicate a corresponding absence of a molecular or transcriptional response (Brady et al. 2011). Examination of such subtle transcriptional changes within individual cell types were first revealed by the use of innovative techniques (fluorescent activated cell sorting, laser capture microdissection, translating ribosome affinity purification, isolation of nuclei tagged in individual cell types) coupled with microarray or RNA sequencing analysis, facilitating transcriptome-scale cell type–specific investigations (Fig. 2) (Birnbaum et al. 2003; Day et al. 2005; Zanetti et al. 2005; Dinneny et al. 2008; Gifford et al. 2008; Deal and Henikoff 2011). These methodologies provided a fundamental framework to understand how plant root cell types respond to diverse factors and transcriptionally integrate these responses (Dinneny et al. 2008; Gifford et al. 2008; Long et al. 2010; Iyer-Pascuzzi et al. 2011).

**Figure 3.**
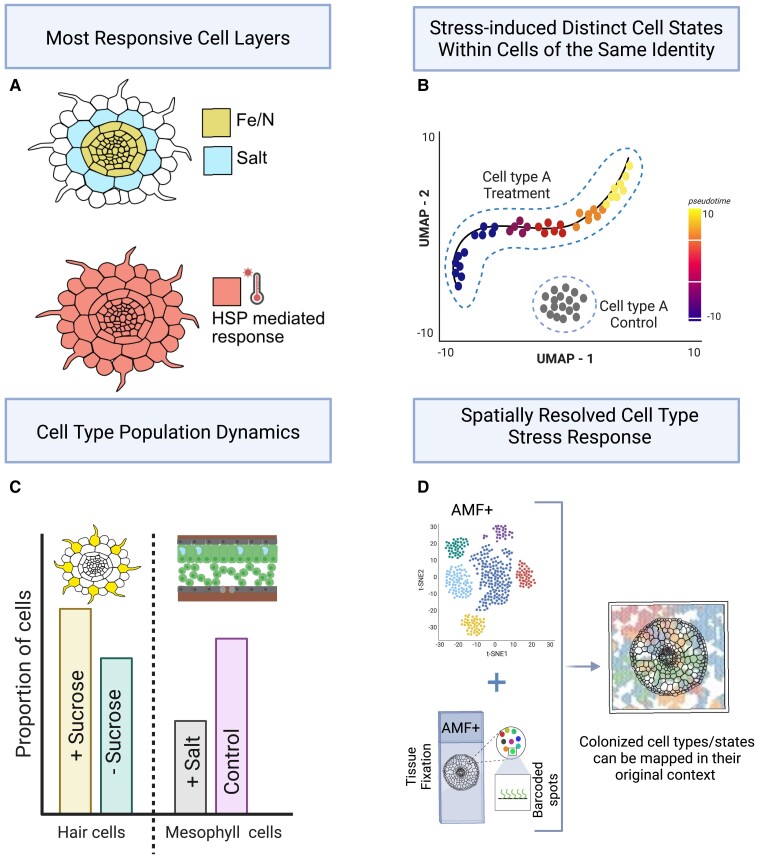
The multifaceted responses of root cell types to stress. **A)** Cross-section diagrams of the Arabidopsis root illustrating the most transcriptionally responsive cell types to specific stressors. The stele is the most transcriptionally responsive cell layer under iron (Long et al. 2010) and nitrogen deficiency (Gifford et al. 2008), while the cortex is the most responsive under salinity (Dinneny et al. 2008). In contrast, heat stress can trigger a universal heat shock protein (HSP)–mediated transcriptional response across all cell layers (Jean-Baptiste et al. 2019). **B)** Schematic of a uniform manifold approximation and projection (UMAP) showing stress-induced distinct cellular states within cells of the same identity (Zhu et al. 2023). **C)** Bar graph depicting the shift in cell population dynamics in response to external stimuli, with an increase in hair cell population with sucrose supplementation (Shulse et al. 2019) and a decrease in mesophyll cell population under sodium stress (Wang et al. 2021). **D)** Integrating snRNA-Seq in root tissue colonized by arbuscular mycorrhizal fungi (AMF+) with spatial transcriptomics enabled mapping of the colonization responses to their original spatial context within colonized root tissue to identify localized and colonization stage-specific transcriptional responses (Serrano et al. 2024). Figure created with Biorender.com.

Before this era, whole root transcriptional studies operated under the assumption that the root was a single unit of transcriptional response (Fig. 2). However, transcriptome profiling of Arabidopsis root cell types grown with a high salt (NaCl) concentration revealed very few genes whose expression significantly changed in all cell layers (Dinneny et al. 2008). Correspondingly, the majority of differentially expressed genes were cell type and developmental zone specific, with the cortex layer being the most transcriptionally responsive, as determined by the number of detected differentially expressed genes (Fig. 3A). Using epidermal-patterning mutants, Dinneny et al. (2008) highlighted the role of cell fate regulators within an individual cell type in response to salt stress. Indeed, there were sets of genes whose differential expression under stress was dependent on correct epidermal specification and patterning (Dinneny et al. 2008). These cortex- and epidermis-specific observations interestingly align with cell layers involved in root halotropism response, where an ABA-activated protein kinase SnRK2.6 drives cortical MT reorientation at the root transition zone to slightly increase volume in the cortex and epidermis of Arabidopsis (Yu et al. 2022). Likewise, iron deficiency elicits cell type or tissue-specific transcriptional responses, with the stele as the most responsive (Dinneny et al. 2008; Long et al. 2010). This observation aligns with nitrogen deficiency responses (Gifford et al. 2008) and is attributed to the stele's critical role as the transport hub of plant roots (Fig. 3A). The significant enrichment of differentially expressed transcription factors within the pericycle during iron deficiency was used to generate a hypothesis that pericycle-specific transcription factors coordinate the iron deficiency response. The transcription factor POPEYE, whose expression is induced under iron deficit, was functionally validated as a regulator of iron homeostasis between the root's outer layers and the stele (Long et al. 2010).

Three key biological insights have emerged from studying the transcriptional behavior of specific root cell types in response to external nutrient stressors. First, there is little evidence for conservation of a universal transcriptional stress response across distinct cell types undergoing the same stress. Although a very minimal shared transcriptional stress response is present, it cannot be generalized across all cell types. Second, the identity of a cell can dictate the specific gene sets that are activated or repressed in response to a particular stress as demonstrated by distinct functional gene categories enriched in each cell type under various stress conditions (Dinneny et al. 2008; Iyer-Pascuzzi et al. 2011). This point is interrelated with the first, as the cell type–specific transcriptional responses are developmentally determined and result in the lack of a universal stress response across different cell types. Further, “response nonredundancy,” where expression of individual transcripts within a functional group is highly cell type specific, enables specialization of cell-type activity while maintaining shared functional responses (Walker et al. 2017). Lastly, a group of developmental regulators appears to maintain stable expression patterns, unaffected by the environment, thereby sustaining cell identity and triggering cell-specific responses to stimuli. Furthermore, there is a portion of the transcriptome that remains nonplastic and conserved in multiple cell types, regardless of environmental stimuli, such as housekeeping genes, which are essential for maintaining basic cellular functions (Reynoso et al. 2022).

### The impact of scRNA-Seq on understanding plant transcriptional responses to stress

In recent years, single-cell RNA sequencing (scRNA-seq) has emerged as a powerful technique in plant research, enabling the capture of transcriptome dynamics of individual cells within a tissue across multiple species, including Arabidopsis, maize, rice, tomato, tobacco, poplar, sorghum, and setaria (Efroni et al. 2016; Shulse et al. 2019; Song et al. 2020; Dorrity et al. 2021; Kim et al. 2021; Ortiz-Ramírez et al. 2021; Seyfferth et al. 2021; Xu et al. 2021; Kang et al. 2022; Shahan et al. 2022; Xie et al. 2022; Guillotin et al. 2023; Lee et al. 2023; Cantó-Pastor et al. 2024). This technique provides unparalleled insight into the transcriptional heterogeneity, or distinct transcriptional states, among cells of the same identity, surpassing the capabilities of earlier methods that isolated entire cell types (Fig. 2). This heterogeneity is particularly relevant when considering cell-specific responses to environmental stress. Traditional methods could not capture these subtle yet significant differences in how individual cells with the same identity may respond differently to a given stimulus. scRNA-seq can overcome this limitation by identifying rare or transient subpopulations of cells with unique molecular signatures that are important to understand a plant's response mechanisms. For example, a group of cells within a tissue may exhibit different transcriptional responses or states to a particular stress compared with identical neighboring cells, a complexity that only scRNA-seq can resolve. Furthermore, the integration of pseudotime algorithms with scRNA-seq data allows reconstruction of developmental trajectories to capture possible stress-induced cellular heterogeneity in a temporal manner (Fig. 3B). An excellent example of single-cell transcriptomic profiling's ability to discern cellular transcriptional heterogeneity is illustrated through the interactions between Arabidopsis and the pathogen *Pseudomonas syringae*. The continuum of disease progression within the leaf was shown to gradually transition from an immune state to a susceptible state during the continuum of infection. Further, some cells were immediately transcriptionally responsive to pathogen invasion, while others responded at a later stage (Zhu et al. 2023).

In contrast to the many cell type–resolution maps of root development, fewer scRNA-seq studies have concentrated on cell-specific transcriptional responses to abiotic stresses (Jean-Baptiste et al. 2019; Wendrich et al. 2020; Wang et al. 2021). Jean-Baptiste et al. (2019) subjected whole seedlings to heat stress and analyzed the outcome using single-cell RNA sequencing. Contrary to the prior observations of little to no whole root transcriptional responses (Dinneny et al. 2008; Iyer-Pascuzzi et al. 2011), this study discovered that canonical heat-shock genes were predominantly differentially expressed across all cell types (Fig. 3A). They also observed cell-specific responses; hair cells showed an enriched response of genes associated with ribosomes and RNA methylation. In contrast, stele cells showed varied expression in genes associated with cell wall organization and biogenesis, while endodermis cells demonstrated distinct expression patterns in genes linked to chemical and stress response stimuli, as well as in nitrate and anion transport. The pan-root transcriptional responsiveness of heat shock genes demonstrates that although most stress-induced responses are cell type specific, this is not always the rule.

Wang et al. (2021) expanded the scope of single cell research to a crop species by examining the transcriptional response of rice seedlings to a broader spectrum of abiotic stress conditions: low nitrogen, high salinity, and iron deficiency. In response to each individual stress, again a significant proportion of differentially expressed genes was within a specific cell type (Wang et al. 2021). Despite this predominant mode of responsiveness, some common responses were also observed—not only in roots but also in leaves. Besides these cell type–specific transcriptional responses, a proportional change in the size of cell populations was also observed. Specifically, a decrease in the mesophyll cell population size was observed under high salinity (Fig. 3C). In contrast, the mesophyll cell population size remained largely unchanged under iron deficiency and low nitrogen (Wang et al. 2021). This suggests a stress-induced adaptation in specification or maintenance of mesophyll cell identity or of cell proliferation. Further probing of the molecular basis underlying this proportional shift indicated that high-salinity treatment altered mesophyll cell expression profiles at different developmental stages, disrupting their normal maturation and reducing the cell population (Wang et al. 2021). A similar phenomenon was observed by Shulse and colleagues (2019) regarding sucrose supplementation of Arabidopsis roots. Here, a strong enrichment of the hair cell population was observed in response to sucrose while there was an enrichment of the meristematic cell population without sucrose (Shulse et al. 2019) (Fig. 3C).

In the context of a plant's response to phosphate deprivation, Wendrich et al. identified the critical role of the TARGET OF MONOPTEROS 5/LONESOME HIGHWAY (TMO5/LHW) transcription factor complex (Wendrich et al. 2020). Through high-resolution single-cell gene expression analysis of Arabidopsis roots, this study demonstrated how the TMO5/LHW complex increases root hair density in phosphate deficiency. This is achieved by altering epidermal cell fate and length, thereby enhancing phosphate foraging efficiency. This highlights a precise cellular adaptation to nutrient stress, with the cytokinin pathway connecting vascular cell perception of phosphate levels to trichoblast differentiation (Wendrich et al. 2020).

One of the advantages of scRNA-seq is the elucidation of changes in transcriptional response in a single cell type's developmental trajectory. In principle, such a response is possible as observed by Dinneny et al. (2008), where in response to salt stress in Arabidopsis, the elongation zone was the most transcriptionally responsive as defined by the number of significantly differentially expressed genes (Dinneny et al. 2008). The changes in meristematic cell population size identified by Shulse et al. (2019) in the absence of sucrose further supports the observation of changes in developmental time. Spatial context is equally important in plant–biotic interaction, where spatially confined damage to specific subset of cells within the root are shown to be sufficient to induce and propagate responsiveness in neighboring nonresponsive cells (Zhou et al. 2020). However, a significant limitation of scRNA-seq is its inability to maintain the spatial context of cells. Once cells are dissociated from their native environment for analysis, crucial spatial information is lost, making it challenging to comprehend how cells interact within their microenvironment and collectively respond to external stimuli.

While the impact of scRNA-seq in plant research is significant, limitations in its widespread adoption across plant species remain. The rigid cell wall in plants varies in composition across cell types, species, and environments and is dynamically modulated in response to environmental stimuli. As in cell type–profiling methods that require fluorescent activated cell sorting, scRNA-seq approaches require cell wall dissociation to release individual cells, known as protoplasting. This process can introduce an extraction bias toward cells that are more amenable to enzymatic digestion or those located on the tissue's outer layers and are more accessible to enzymes, potentially skewing the representation of certain cell types or developmental stages. Moreover, the enzymatic digestion process itself can result in stress-induced transcriptional responses and data artifacts that can in part be resolved by identifying protoplasting-induced genes and removing these from future analyses (Birnbaum et al. 2003; Cantó-Pastor et al. 2024). If these protoplasting-induced genes are also important for a cell type response, however, then they would not be identified. Plant cell size diversity also brings another layer of complexity to single-cell analysis, as microfluidic platforms require some uniformity in cell sizes (Whitesides 2006). The stringent requirement for a high quality and quantity of protoplasts extracted, along with the high costs of specialized reagents and instruments, further limits this technique's applicability across plant species and laboratories.

Single-nucleus RNA-seq (snRNA-seq) is an alternative for single-cell transcriptomics in plants through isolated nuclei, offering added advantages for studying fixed or frozen samples without the need for protoplasting (Farmer et al. 2021; Marand et al. 2021; Neumann et al. 2022). These are particularly of use in studies focused on plant responses to external stimuli, where the risk of triggering protoplasting-induced stress responses similar to those being investigated are eliminated. This approach further broadens the range of plant species, cell types, and cell wall–based transcriptional reprogramming that can be analyzed. However, there are trade-offs; nuclear transcripts often represent a fraction of transcriptome in a cell, thus limiting the capture of cytoplasmic transcripts or those with less nuclear abundance. In line with this, the average number of genes detected in single-nuclei profiling studies can be significantly lower compared with those identified in single cells (Guillotin et al. 2023). Additionally, single-nuclei datasets tend to produce fewer distinct cell clusters and often struggle to differentiate between closely related or subcellular identities (Guillotin et al. 2023).

Benchmarking of single-cell relative to single-nuclei approaches is reviewed and extensively described in Grones et al. 2024 (Grones et al. 2024). Numerous platforms for both single-cell and single-nucleus profiling are also available, and combinatorial barcoding approaches effectively overcome the scalability and cost limitations inherent to microfluidic methodologies (reviewed in Grones et al. 2024). In combinatorial barcoding, each cell's mRNA are uniquely tagged through multiple rounds of barcoding, allowing for high-throughput analysis and sample multiplexing without complex equipment. This approach allows for the simultaneous analysis of large numbers of samples and nuclei, making large-scale projects possible and cost-effective. Additionally, the ability to use fixed samples makes the protocol highly flexible and enhances scalability. Although this approach remains to be widely adopted in plant research, it has been successfully applied in profiling chromatin accessibility at single-cell resolution in Arabidopsis (Tu et al. 2022).

Spatial transcriptomics is a complementary technology to sn/scRNA-seq by preserving the spatial context of transcriptional profiles within tissues. This technology can pinpoint specific zones within a cell type where stress responses are initiated and how these signals propagate, potentially offering a 3-dimensional perspective on stress response. Despite its promise, spatial transcriptomics is still in infancy in plant biology research, and it has been utilized in only a handful of studies focusing mostly on plant development or biotic interactions (Moreno-Villena et al. 2022; Liu et al. 2022a, 2022b; Xia et al. 2022; Nobori et al. 2023; Serrano et al. 2024).

A recent pioneering work combining these complementary approaches—snRNA-seq and spatial RNA-seq—elucidated the complex nature of interaction between the *Medicago truncatula* root and a symbiotic partner, the arbuscular mycorrhizal fungus (AMF) *Rhizophagus irregularis*, in a spatially resolved fashion (Fig. 3D) (Serrano et al. 2024). Spatial transcriptomics allowed for simultaneous gene expression analysis of both the plant and AMF in the colonized root zones, identifying clusters in the spatial dataset with high expression levels of known colonization stage-specific genes overlapping with AMF-responsive zones (Serrano et al. 2024). Fungal expression was also correlated with the presence of arbuscules—branched structures formed by the differentiation of fungal hyphae within the root cortical cells, which are central to the nutrient and water exchange in the symbiotic relationship between AMF and plants. The spatial dynamics of the symbiosis were mapped across individual root cross-sections by tracking the distribution of marker genes indicative of early to late-stage colonization. SnRNA-seq further identified a distinct “colonized cortex cell cluster,” which, when integrated with the spatial dataset, discovered hundreds of novel AMF-responsive *M. truncatula* genes that could serve as a great resource for further research (Serrano et al. 2024). As of yet, there are no published studies to our knowledge that have adopted a similar complementary approach with respect to plant-abiotic factor interactions.

### Conclusion and perspectives

The plant root system performs a multitude of critical functions, from nutrient uptake to interactions with the surrounding soil environment. Roots adapt dynamically to various challenges, including biotic and abiotic stresses, such as microorganisms, drought, and salinity, by altering their system and cellular architecture as survival strategies. Each cell type within the root can exhibit unique responses, dependent on the stimulus as well as the species, indicative of an evolutionarily derived adaptability (Fig. 1). The molecular mechanisms underlying these adaptive changes have been increasingly elucidated through cell type–specific transcriptional methodologies. Our understanding of stress response in plant roots is now recognized as the sum of cell type–specific responses (Dinneny et al. 2008; Gifford et al. 2008; Long et al. 2010; Iyer-Pascuzzi et al. 2011; Jean-Baptiste et al. 2019; Wendrich et al. 2020; Wang et al. 2021; Zhu et al. 2023; Serrano et al. 2024). The significance of cell identity in mediating stress responses is increasingly acknowledged, emphasizing the need for further exploration of how known cell identity regulators function within stress-responsive pathways. Observations of changes in the size of specific cell populations under stresses like heat or salinity in Arabidopsis roots and rice leaves point to dynamic shifts that occur in cell type differentiation, though the mechanisms and physiological implications of these changes are not yet fully understood (Dinneny et al. 2008; Iyer-Pascuzzi et al. 2011; Jean-Baptiste et al. 2019; Wendrich et al. 2020; Wang et al. 2021).

Integration of advanced omic tools in studying root–environment interactions is critical to further advancing this understanding. Spatially resolved, cell-specific transcriptional maps can elucidate complex local intercellular communications when facing environmental stress. Expanding this approach beyond the model plant Arabidopsis to include other species will enable utilization of the extensive genetic (both mutant and population-scale) resources of diverse plant species (both stress tolerant and susceptible), which is crucial to elucidate their tolerance mechanisms and to inform breeding strategies for stress resilience. Furthermore, it is essential to address the complexity of real-world conditions where plants often simultaneously face multiple stresses, such as drought combined with high temperatures or salinity stress. Understanding how transcriptional profiles of individual cell types are reshaped when navigating these multi-stress environments is crucial for developing crops that can withstand such conditions, ensuring agricultural sustainability in our changing climate.

**Advances Box**

- **Root cell type–specific transcriptional responses:** Emerging research has elucidated the unique ways in which individual root cell types of plants react to environmental stresses. This shift from viewing the root as a homogenous response unit to recognizing the cell-specific responses offers a deeper understanding of plant resilience and adaptation. Most, but not all, responses are divergent across cell types.
- **Breakthrough techniques for cell-specific analysis:** Advanced techniques such as single-cell or nucleus RNA sequencing have enabled, at an unprecedented resolution, the study of how individual cell types within plant roots respond to various external factors.
- **Understanding cell identity and stress response:** The identity of a root cell plays a crucial role in dictating its transcriptional response to stress. This insight emphasizes the importance of developmental regulators in maintaining cell identity under stress, enabling precise, cell-specific responses to environmental challenges.
- **sn/scRNA-seq insights:** sn/scRNA-seq has emerged as a key tool in plant research, offering insights into distinct transcriptional states of cells with the same identity, especially in their response to environmental stress, revealing complexities previously impossible with bulk analyses.
- **Spatially resolved transcriptomics:** Combining cell-specific RNA sequencing with spatial transcriptomics has provided a spatial map of specific plant root zones and cell types that interact with symbiotic partners, underscoring the potential of these technologies to reveal novel aspects of root cell type responses to external factors.

**Outstanding Questions Box**

- How do cell identity regulators facilitate stress responses within a specific cell population of a given cell type?
- What mechanisms underlie the dynamic shifts in cell type differentiation observed in plant roots under various stresses?
- What are the physiological implications of morphological and molecular changes within individual cells?
- What cell-specific mechanisms enable stress-tolerant species to survive adverse conditions?
- How do simultaneous multiple stresses impact the cell-specific transcriptional responses in crops?

## Dive Curated Terms

The following phenotypic, genotypic, and functional terms are of significance to the work described in this paper:

root AmiGo: PO:0009005

xylem AmiGo: PO:0005352

PIN2 Gramene: At5g57090
PIN2 Araport: At5g57090

cortex AmiGo: PO:0005708

epidermal cell AmiGo: PO:0004013

lateral root AmiGo: PO:0020121

metaxylem AmiGo: PO:0000372

VND6 Gramene: At5g62380
VND6 Araport: At5g62380
