# Correction to: The transcriptional integration of environmental cues with root cell type development

**DOI:** 10.1093/plphys/kiaf028

**Published:** 2025-02-13

This is a **correction** to:

Mona Gouran, Siobhan M Brady, The transcriptional integration of environmental cues with root cell type development, *Plant Physiology*, Volume 196, Issue 4, December 2024, Pages 2150–2161, https://doi.org/10.1093/plphys/kiae425

In Figure 1 of the originally published version of this article, the labels ‘PERICYCLE’ and ‘ENDODERMIS’ were inadvertently switched.

This error has been corrected.

